# Exploratory content analysis of direct-to-consumer pet genomics: What is being marketed and what are consumers saying?

**DOI:** 10.1371/journal.pone.0261694

**Published:** 2022-01-07

**Authors:** Nikki E. Bennett, Silvio Ernesto Mirabal Torres, Peter B. Gray

**Affiliations:** Department of Anthropology, University of Nevada, Las Vegas, Nevada, United States of America; Universidad Internacional de La Rioja, SPAIN

## Abstract

Mars Petcare introduced the first direct-to-consumer domestic dog genetic test in 2009 and Basepaws introduced the first direct-to-consumer cat genetic test in 2016. Social science research has evaluated numerous aspects of the human direct-to-consumer market, yet no such exploration has evaluated the occurrence of pet owners pursuing pet genetic tests. Using a mixed methods approach, we conducted an exploratory content analysis of direct-to-consumer pet genetic company webpages and consumer reviews shared on Amazon. Initial data reviews indicated some companies may be key industry players, relative to others. Our results present content frequency for each group (key industry players, all other companies), though the primary themes for each remained the same. Analysis showed genetic companies are primarily sharing product and purchasing information, along with trustworthiness to establish the merit of the company and their products. Companies also used statements directed towards pet owners that are suggestive of both pets and “pet parents” benefiting from the test results. The primary themes identified in consumer reviews involved consumers sharing their perception about the tests (e.g., accuracy), what aspects of the test results they focused on (e.g., breed information), and experiences with using the test (e.g., ease of use). Amazon reviews were primarily positive, though the companies with smaller review numbers had higher percentages of negative and ambiguous sentiments. Of interest, reviews most often indicated tests were being used to determine a pet’s breed identity, while companies most frequently promoted the health advantages of using their products. Reviews revealed some consumers respond to tests by sharing their pet’s results with someone or by altering their pet’s care. Considering these results in addition to the growing popularity of this industry and the advancements of genomic technology, further research is needed to determine the role pet genetic testing may have in society and on human-animal relationships.

## Introduction

The personal genomic industry has skyrocketed since the human, dog, and cat genomes were sequenced in the first decade of the 21^st^ century. The global market for human genetic testing is projected to reach $28.5 billion by 2026 [1] and the animal genetic market was valued at $990 million in 2020 [2]. Mars Petcare developed the first canine DNA test in 2007, requiring a veterinarian to perform in clinical settings [3]. Following in the steps of the human market, Mars moved to a direct-to-consumer (DTC) platform citing veterinarians as a hinderance to high consumer demands for pet DNA tests [4]. The social and ethical implications for both the human and pet DTC genetic industries are controversial, though research has primarily focused on the human-use market. The scholarly literature on DTC pet genomics consists of commentary or theoretical perspectives (e.g., [5]). The present study utilizes current research on human-pet dynamics and the DTC human genetic test market to inform the theoretical framework and make inferences about the parallels between human and companion animal DTC genetic testing (DTC-GT).

### Pet owners and “Dr. Google”

The American Pet Products Association [6] estimates U.S. pet owners spent approximately $103.6 billion on their pets in 2020, with 30% of this amount going towards veterinary care and “products.” Despite trends of American pet owners spending more money on their pet’s care, it is well known that pet owners consult “Dr. Google” (i.e., search the Internet) for veterinary care information (e.g., [7]). A survey conducted in the U.K. reported participants turned to the internet more than a veterinarian for pet health information [8]. Another survey distributed to U.S. veterinary clinics and their clients found 73% of pet owner participants used the internet for veterinary care information [9]. Similarly, interviews with U.S. veterinarians and pet owners have also indicated pet owners rely more on Internet information than their veterinarian [10].

Another sign that pet owners may avoid taking their pets to their veterinarian is pet poison statistics. According to the American Society for the Prevention of Cruelty to Animals’ (ASPCA) poison help-line [11], human medications rank as the highest toxicant ingested by pets annually with over-the-counter (OTC) medications making up 20% of their caseload in 2019. Though the ASPCA does not share data on how pets encountered human medications, pet insurance companies have reported pet owners intentionally administer human OTC medications without knowing correct doses or if the medicine is toxic to nonhuman animals [12]. These tendencies for pet owners to use other sources for pet medical information instead of consulting a veterinarian are relevant to the DTC pet genetic industry, especially with tests becoming more advanced and offering health-related information.

### Pet genomic results: Knowing just enough to be dangerous

#### Health results

Similar to human testing, pet genetic health results can provide pet owners with information about their pet’s genetic predisposition towards diseases, carrier status of deleterious genes, and other related medical information (e.g., medicine sensitivity, immune response). However, it is unknown how pet owners interpret this information and behaviorally respond to pet health results (e.g., consult a veterinarian). Turning to what is known about the human DTC-GT industry, research has found consumers understand and react to results differently. Kaufman et al. [13] reported consumers had health-related behavior changes (e.g., seek additional information, change medications) after using DTC-GTs. These behavioral responses were related to the consumer’s subjective interpretation of the test results (e.g., family history of a disease). In considering consumer test interpretation, Ostergren et al. [14] determined consumer comprehension was related to demographics (e.g., age) and education. Specifically, study participants were better at interpreting drug response and health summary reports but did not perform as well when deciphering information about specific carrier status [14]. Supporting what others had already expressed concerns about, these studies show how DTC-GT can have warranted or unwarranted psychological and behavioral impacts [15].

Although pet health information from DTC-GT can be helpful and potentially improve pet health outcomes, a real-life case study has illuminated the ethical dilemma of pet owners pursuing and interpreting genetic test results without consulting a veterinarian. After showing symptoms of incontinence and neurological symptoms, a dog owner purchased a DNA test and received results indicating her dog was a carrier for Degenerative Myelopathy (DM), a neural disease. Based on these results, the owner had her dog humanely euthanized [5]. Commenting on this case study, experts say the prognosis of dogs carrying the mutation for DM is difficult to interpret, with carriers possibly never showing symptoms [4]. This case demonstrates the need to understand how pet owners interpret their pet’s test results.

#### Breed results

In addition to health information, personal pet genomics return information about pet ancestry (e.g., breed information, multi-generational pedigrees) and physical traits (e.g., coat color and length). At present, the American Kennel Club (AKC) [16] recognizes 197 dog breeds and the Cat Fanciers’ Association (CFA) [17] recognizes 45 cat breeds. The phenotypic consistencies observed between “breeds” represent human induced artificial selection and geographical influences [18]. The operationalization of breed classification has social implications in which particular breeds are stereotyped behaviorally and associated with particular human demographics. Breed-specific legislation (BSL), also referred to as “Dangerous Dog Breed Laws,” is controversial with the American Veterinary Medical Association (AVMA) [19] openly criticizing its efficacy to prevent dog attacks. BSL is problematic as discerning “dangerous” dogs from “non-dangerous” dogs relies on unreliable visual identification (e.g., [20]).

Dangerous dog discrimination is not limited to the dogs themselves and, by default, pet owners with “dangerous looking dogs” are also discriminated against. Fiala [18] argues racism against human demographics is mirrored in “breedism.” The categorization of dogs as “Pit bulls” is one such example. Pit bulls are not an actual dog breed but rather a colloquial categorization applied to particular dogs with certain physical features (e.g., large heads) often found in bull terriers (e.g., American Staffordshire Bull Terrier). In addition to Pit bulls being generalized based on physical appearance, they are associated as being aggressive and owned by marginalized groups of people [21]. Considering these stereotypes in addition to indirect prejudice against pet owners, one must consider DTC pet genetic testing’s potential role in society and/or breed-specific legislation.

#### The present study

As the background above indicates, empirical research is needed to evaluate why consumers purchase pet genetic tests and how they interpret the returned results. Furthermore, pet genomics as “big business” raises concerns about the industry’s efficacy, trustworthiness, and motivations (see [5] for further detail). To develop a comprehensive understanding of the products being marketed and consumer experiences, this study entailed an exploratory thematic analysis of the pet genetic industry. Our first objective was to evaluate genetic company websites and make determinations about the information being shared and how pet genetic tests are marketed to consumers. The second objective was to explore pet owner experiences with getting their pet genetically tested. To meet these aims, we first identified companies in the DTC pet genomics industry. Then, using a mixed-methods approach, we completed a content analysis of genetic test company websites and publicly available consumer reviews. In line with a content analysis, we present the primary themes in the results for all genetic companies. As findings below reveal, there appear to be three major DTC pet genetic key industry companies–Basepaws, Embark, and Wisdom Panel–as indicated by the number of consumer reviews. Therefore, in addition to providing the primary themes and code frequencies, we provide additional context to the three key perceived industry leaders and the remaining companies. Considering the exploratory nature of this project, this study incorporated a grounded theory approach in which data was collected and analyzed with the intent to develop follow-up study protocols and research questions. As such, no hypotheses were formally tested.

## Methods

This study followed an exploratory thematic analysis protocol, which is useful for identifying themes (also referred to as codes) present in particular content. Thematic analyses also allow for inductive approaches (discussed further below) and the integration of grounded theory (see [22] for further reference).

### Sample identification

Between December 2020 and February 2021, an exploratory Internet search was used to establish genetic companies advertising pet genomic services. Eleven private sector companies were identified, but certain companies were excluded from analysis. AKC testing services was removed as the test offered only established pedigree (i.e., parentage) and explicitly stated pet owners should not use the test for any other use. Another company, Neogen, was removed due to their strict marketing and distribution to veterinarians. The Europe-based company, MyDogDNA, was also excluded due to all other companies and Amazon consumer reviews included in this study being US-based. After careful review, we identified eight pet genetic companies to use for this analysis (Table 1).

**Table 1:** Direct-to-consumer nonhuman animal genetic testing services identified using general internet search query. Using the company’s descriptive language, details include the tests offered, species the test is used for, and what the test was advertised to include.

Company Name	Tests Offered	Species	Test Includes
Basepaws	Breed + Health DNA Test	Feline	Breed groups, health, traits, and habits.
Canine HealthCheck	Canine HealthCheck Kit	Canine	Health, phenotype/genotype
DNA My Dog	DNA My Dog NEXTGEN Breed Identification and Genetic Age Test	Canine	Breeds, personality traits, genetic health concerns, predisposition to disease
EasyDNA	Premium Dog Testing Package	Canine	Dog allergy test, breed test, genetic age test
EasyDNA	Cat Genetics DNA Test	Feline	Breed similarities, wild cat index percentage
Embark	Breed + Health Kit	Canine	Health conditions, physical traits, breeds, family tree, relatives
Optimal Selection	Optimal Selection Canine	Canine	Health, Traits Test, Breed, Genetic Diversity
Optimal Selection	Optimal Selection Feline	Feline	Health, Traits
Orivet Genetic Pet Care	Genopet 5.0 (Breed + Health Kit)	Canine	Breeds, Health, LifePlan
Orivet Genetic Pet Care	Cat DNA Health Screen	Feline	Health, LifePlan
Wisdom Panel	Wisdom Panel Premium	Canine	Genetic conditions, breeds, ancestry, physical traits, veterinarian consult

### Data collection

Website data were collected between January and March 2021. To preserve the genetic company webpage data, in addition to the stored PDF files, Internet Archive Wayback Machine was used. All websites were saved as PDF text files and imported into ATLAS.ti 9 for iOS operating system. Due to the differences in genetic company website layouts (e.g., blog sections, research information subpages), we only used the main homepage that appears when the official website URL is used and test product information pages. If a company marketed more than one test for the same species (e.g., Wisdom Panel Premium vs. Wisdom Panel Essential), then the most comprehensive test was used for analysis. This also provided consistency in data processing as certain companies offered tests individually or as a package deal (e.g., EasyDNA). Some companies offered genetic testing for other species (e.g., avian); however, only data related to canine and feline tests were used in this analysis.

Several inclusion and exclusion criteria were applied during e-commerce sample selection. At the time of data collection, not all tests were marketed equally. This was evident with test purchase options being limited to distribution from the company directly or the availability of tests on third party markets. For example, Orivet’s Genopet 5.0 test was available on Amazon, Chewy, eBay, Petco, and PetSmart while EasyDNA could only be purchased through their website. Another challenge was not all tests were available on the same third party markets (e.g., Basepaws was only available on Amazon, but Embark available on Chewy and Amazon). For all tests that were available through third party purchase, Amazon was always used and, therefore, was the only e-commerce platform used to evaluate consumer reviews. As previously mentioned, and reflected in Table 2, certain companies exclusively sold their tests and did not offer their products on third party markets. Therefore, no consumer reviews were available to use for these companies.

**Table 2:** Final sample for each genetic company by number of homepages, test pages, and Amazon reviews.

Company Name	Homepage (#)	Species	Amazon Reviews (#)	Genetic Test Page (#)
Basepaws	1	Feline	100	1
Canine HealthCheck	1	Canine	3	1
DNA My Dog	1	Canine	34	1
EasyDNA	1	Canine	0	1
		Feline	0	1
Embark	1	Canine	100	1
Optimal Selection	1	Canine	0	1
		Feline	0	1
Orivet Genetic Pet Care	2	Canine	35	1
		Feline	14	1
Wisdom Panel	1	Canine	69	1

Lastly, unequal notoriety for tests had to be considered. A general Internet search provides several pages discussing the “top dog [or cat] DNA tests” (e.g., [23]). This was worth considering as these tests have upwards of 5000 reviews on Amazon alone (e.g., Wisdom Panel), while other companies (e.g., Orivet) had much smaller review sets to sample from and the majority of these reviewers received an incentive to do so (see Reward for Review code in Table 4). To best account for popularity and time on the market, we collected the 100 most recent reviews with the following Amazon filter: Most recent, All reviewers, All stars, All formats, and Text, image, video. Amazon allows consumers to share photographs on review posts, but they were not included in this analysis. Table 2 provides a breakdown of the final consumer review sample.

#### Data collection limitations

During analysis and results write-up, it was realized that data collected to evaluate consumer experiences (i.e., Amazon reviews) were more representative of three genetic test companies. Even further, despite Amazon having more than 100 reviews for particular companies, the website would not display all of the reviews. This is reflective of the website’s algorithms that selectively provide materials to users [24]. Unfortunately, we were unable to bypass these algorithms and collect more consumer reviews from Amazon’s platform. In addition to not being able to get all reviews to generate, the filters applied did not maintain timeline uniformity as intended. The use of the “Most recent” filter was used to maintain consistency in the timelines for consumer reviews since tests became available to consumers at various times. However, timeline coherence for reviews only applied to the companies with more than 100 reviews. The implications of these limitations are considered more in-depth in the discussion section.

### Codebook development

Code development and application followed a mixed methods approach. The lead author established the codebook using a sample subset that consisted of Wisdom Panel, Basepaws, and Orivet Genetic Pet Care associated data. This comprised both homepages, product pages, and 20 Amazon reviews for each. An inductive approach was used to identify primary themes and associated subthemes. This also entailed reviewing the subset sample multiple times and coding based on content alone and not with a preconceived list of themes [22]. Then, the initial codebook was reviewed by the other authors for overlapping themes and/or associated code groups to ensure clarity.

To determine the codebook’s validity, an Inter-Coder Agreement (ICA) protocol was followed. RStudio [25] was used to generate a random sample for the ICA which was representative of both genetic company websites and Amazon reviews (10%). ATLAS.ti’s ICA feature was used to calculate reliability using the lead and second author’s application of codes to the ICA subset sample (Krippendorff’s cu α_Amazon Reviews_ = 0.83; Krippendorff’s cu α_Genetic Company Webpages_ = 0.75). The finalized coding scheme with associated definitions are provided in Tables 3 and 4. Once the codebook was verified for reliability, the remaining 18 webpages and 352 Amazon reviews were divided between the first and second author for analysis.

**Table 3:** Codes applied to genetic company webpage and test production information page.

Themes	Description		Example
Credentials	Information related to the company’s accreditation.		.* *.* *.*developed by veterinarians in partnership with Cornell University*.
Endorsement	An expert, celebrity, or other outside source providing approval or support of the genetic test company or pet genetic testing.		*In the press*: *“The tests are comprehensive*.* *.* *.* *.*giving us faith that we could trust the test outcomes*.*”*
Information	Instructions	Company provides information about the genetic test and its associated features; how to use kits; and/or information related to its cost or how to purchase.	*$159*.*99 Screens for 200+ genetic conditions*, *350+ breeds*.* *.* *.
	Process		
	Product		
	Purchasing		
Learn About Pet	Company advertising the advantages of using their product(s) for consumers to learn about their pet.		.* *.* *.*discover more about what makes your furry companion the way they are*
Personalized Test Results	Company advertises the tests as offering idiosyncratic information about a consumer’s pet. Examples include personalized genetics, customized wellness plans that are derived from the advertised product and the pet’s individual genetic make-up.		.* *.* *.*includes a personalized LifePlan*.* *.* *.
Photograph	Canine	Professional photograph used for marketing on company webpage.	−
	Feline		
	Human-Animal Interaction		
Promotion	Company provides a link to another subpage and/or a "promo code" for the consumer to get a discounted rate.		.* *.* *.*$40 off a Breed + Health Kit*.
Results	Ancestry	Statements about what type of test results will be provided when using the genetic test.	.* *.* *.*full breakdown of your dog’s breed mix*.* *.* *.* *.
	Breed		
	Health		
	Phenotype/Traits		
	Whole Genome		
Services	Information about additional products and/or benefits included with purchasing a genetic test from the particular company.		*Chat Live to our DNA Consultant*.
Stay Connected	Link or form for consumer to enter contact information for the company to send them additional information. Also includes links to social media pages.		*Sign up for our newsletter*.
Technology & Methods	Information promoting the science for their tests and how the results are derived.		.* *.* *.*genetic information with high coverage (>15X) WGS*.
Trustworthy	Indicator that consumers can trust the company and their services. Examples include companies stating they care about their customers, assurance test results have merit and other qualities to convey consumers can be confident in the company and its products.		*Results you can trust*.
Video	Video clip on genetic company web page to provide some sort of product information or demonstrate how consumers process their kits (e.g., collect sample, send in for analysis).		−
Well-Being	Statements about the use of their products to promote health and happiness of pet owners and/or pets.		.* *.* *.*products have enhanced the lives of pet parents just like you*.

**Table 4:** Codes applied to Amazon reviews.

Themes	Description		Example
Behavioral Response	Altered Pet Care	Consumer takes an active response to their pet’s test results.	.* *.* *.*helped me with an immediate issue and was helpful when talking with my veterinarian*.
	Shared Results		
	Other		
Consumer Experience	Easy to Use	Consumer provides context to their experience using the product.	*Collecting the DNA sample was easy*.* *.* *.* *.
	Fun		
	Not Easy to Use		
	Other		
Cost of Test	Complaint	Consumer shares sentiment about the monetary value of the genetic test.	.* *.* *.*worth the money*.* *.* *.* *.
	Great Value		
Customer Service	Negative Interaction	Consumer shares their experience with the genetic test company’s customer service.	*When I lost my first kit in the mail they sent me another one free of charge*!
	Positive Interaction		
Expectations	Expected Results	Pet owner indicates they received (un)anticipated genetic results for their pet.	*It delivered the results I expected plus answered questions*.
	Unexpected Results		
Interpretation	Behavior	Consumer indicates their use of the genetic test to interpret behavioral and/or health outcomes; breed composition; or other outcomes not related to breed, behavior, or health.	*I felt like the kitten had all the characteristics of a Russian blue but couldn’t be sure unless I got the test*.
	Breed		
	Health		
	Other		
Motivation	Education	Consumer gives context to their reason for purchasing a genetic test for their pet.	*I wanted to see what possible medical issues I might face in the future due to genetics*.
	Entertainment		
	Other		
Needs Improvement	Consumer makes a general statement about the company, product, and/or process needing to be revised.		*The product needs serious help with their website*.
Peace of Mind	Consumer indicates the pet genetic testing gave them solace or reassurance.		.* *.* *.*gave me peace of mind that he has no major genetic health issues for me to worry about*.
Perception	Accurate	Consumer shares their personal understanding or sentiment about the company and/or the genetic test.	*Results seem very accurate to me*.
	Confusing		
	Inaccurate		
	Informative		*The health information was very helpful*.
	Not Informative		
	Other		
Recommendation	Another Company	Consumer endorses the company they are reviewing or another pet genetic company.	.* *.* *.*test kit is good overall and I recommend it*.
	Reviewed Company		
Receiving Results	Fast	Consumer comments on the time it took for them to receive test results after submitting their pet’s sample.	*Faster results than when I did my own DNA test*.* *.* *.* *.
	Still Pending		
	Slow		
Review	Ambiguous	The sentiment expressed in the title of the consumer’s review, independent of the complete review itself.	Review Title: *Accurate*, *fast*, *no easy way to share the report*
	Mixed		
	Negative		
	Positive		
Reward for Review	Amazon label for a review completed by a consumer who received an incentive to do so.		*Vine Customer Review of Free Product*
Shared Information	About Pet	Consumer shares information related to their pet’s demographics and/or their pet’s genetic test results.	*I tested a shelter puppy I adopted*. *They guessed she was*.* *.* *.* *.
	Results		
Tested Accuracy	Consumer shares some action they took to make a determination about the accuracy or trustworthiness of the company being reviewed.		*Tested same cat twice*, *different results*.

### Code application & data analysis

Data analysis was conducted between March and April 2021 using ATLAS.ti version 9. After the codebook was established, codes were deductively applied to the remaining dataset and applied within the context of the first objective (i.e., How are pet genetic tests being marketed to consumers?) and second objective (i.e., What experiences are consumers reporting on e-commerce platforms?). There were no limits to how many times a code was applied for website data as themes occurred more than once for different quotes and were positioned at different locations on the same webpage. For example, product information may have been shared at the top and bottom of the web page with other themes separating them (e.g., photographs). As such, this would represent the same theme occurring more than once in the same document as two separate quotes. If content were grouped together in the same location on the webpage, then it was labeled as one quote and relevant codes were assigned. Following this logic, Amazon reviews were assigned their respective codes only once and were grouped according to the user. For example, a consumer may have shared information about the pet they had tested, shared the results of the pet, and then returned to providing additional information about the pet. In this case, the code “shared information about pet” and “shared information about test results” would have only been applied once.

Once each coder had independently completed their analysis, the projects were merged into a larger dataset and collectively reviewed for primary themes in ATLAS.ti. The following results report the primary themes and code frequencies derived from the first and second authors’ code application. The discussion section is derived from all authors’ joint interpretation of those results and details potential follow-up studies and analyses by using the results as a theoretical framework.

## Results

Each results subsection provide a brief discussion about differences between genetic companies that may be perceived as key industry agencies and those who are not. This determination was based off initial exploratory steps to find pet genetic companies (e.g., [23]) and the representativeness of each company in the consumer review sample. Following these discussions, quantitative frequency data is then discussed based on the collective frequencies of all companies (Tables 5 & 6) as, despite possible differences in company success, the primary themes for each remained the same.

**Table 5:** Code frequencies for genetic company webpage content.

Theme	Basepaws, Embark, Optimal Selection, Wisdom Panel		Canine HealthCheck, DNA My Dog, EasyDNA, Orivet Genetic Pet Care		Totals for all Genetic Companies	
	Absolute *f*	Relative *f* (%)	Absolute *f*	Relative *f* (%)	Absolute *f*	Relative *f* (%)
**Information**						
Instructions	7	1.78	9	2.23	16	2.01
Process	8	2.03	17	4.22	25	3.14
Product	29	7.36	39	9.68	68	8.53
Promotion	14	3.55	3	0.74	17	2.13
Purchasing	25	6.35	34	8.44	59	7.40
Services	22	5.58	39	9.68	61	7.65
Total	105	26.65	141	34.99	246	30.87
**Type of Results**						
Ancestry Results	12	3.05	10	2.48	22	2.76
Breed Results	31	7.87	31	7.69	62	7.78
Health Results	42	10.66	39	9.68	81	10.16
Personalized Test Results	14	3.55	17	4.22	31	3.89
Phenotype/Trait Results	16	4.06	15	3.72	31	3.89
Whole Genome Results	4	1.02	0	0.00	4	0.50
Total	119	30.20	112	27.79	231	28.98
**Trust**						
Credentials	5	1.27	6	1.49	11	1.38
Endorsement	18	4.57	12	2.98	30	3.76
Technology/Methods	25	6.35	20	4.96	45	5.65
Trustworthy	15	3.81	21	5.21	36	4.52
Total	63	15.99	59	14.64	122	15.31
**Marketing**						
Photograph Canine	21	5.33	12	2.98	33	4.14
Photograph, Feline	10	2.54	6	1.49	16	2.01
Photograph, Human & Animal	13	3.30	3	0.74	16	2.01
Share Information	2	0.51	5	1.24	7	0.88
Stay Connected	11	2.79	19	4.71	30	3.76
Video	3	0.76	1	0.25	4	0.50
Total	60	15.23	46	11.41	106	13.30
**Targeted Statements**						
Learn About Pet	26	6.60	29	7.20	55	6.90
Well-Being	21	5.33	16	3.97	37	4.64
Total	47	11.93	45	11.17	92	11.54

**Table 6:** Code frequencies for Amazon consumer reviews.

Theme	Basepaws, Embark, Wisdom Panel		Canine HealthCheck, DNA My Dog, Orivet Genetic Pet Care		Totals for all Genetic Companies	
	Absolute *f*	Relative *f* (%)	Absolute *f*	Relative *f* (%)	Absolute *f*	Relative *f* (%)
**Perception**						
Accurate	82	4.52	17	3.17	99	4.21
Confusing	4	0.22	4	0.75	8	0.34
Inaccurate	35	1.93	23	4.29	58	2.47
Informative	142	7.82	29	5.41	171	7.27
Not informative	9	0.50	12	2.24	21	0.89
Other	113	6.22	23	4.29	136	5.78
Total	385	21.20	108	20.15	493	20.96
**Interpretation**						
Behavior	32	1.76	3	0.56	35	1.49
Breed	163	8.98	47	8.77	210	8.93
Health	101	5.56	30	5.60	131	5.57
Other	71	3.91	23	4.29	94	4.00
Total	367	20.21	103	19.22	470	19.98
**Consumer Experience**						
Easy to use	131	7.21	34	6.34	165	7.02
Fun	60	3.30	17	3.17	77	3.27
Not easy to use	5	0.28	19	3.54	24	1.02
Other	39	2.15	10	1.87	49	2.08
Total	235	12.94	80	14.93	315	13.39
**Shared Information About. . .**						
About Pet	126	6.94	29	5.41	155	6.59
Results	92	5.07	30	5.60	122	5.19
Total	218	12.00	59	11.01	277	11.78
**Receiving Results**						
Fast	65	3.58	9	1.68	74	3.15
Simple Statement	27	1.49	6	1.12	33	1.40
Slow	20	1.10	10	1.87	30	1.28
Still Pending	11	0.61	24	4.48	35	1.49
Total	123	6.77	49	9.14	172	7.31
**Motivation**						
Education	76	4.19	13	2.43	89	3.78
Entertainment	9	0.50	1	0.19	10	0.43
Other	27	1.49	2	0.37	29	1.23
Total	112	6.17	16	2.99	128	5.44
**Recommendation**						
Another company	4	0.22	12	2.24	16	0.68
Reviewed company	75	4.13	13	2.43	88	3.74
Total	79	4.35	25	4.66	104	4.42
**Expectations**						
Expected Results	26	1.43	3	0.56	29	1.23
Unexpected Results	57	3.14	16	2.99	73	3.10
Total	83	4.57	19	3.54	102	4.34
**Cost**						
Complaint	17	0.94	21	3.92	38	1.62
Great Value	39	2.15	6	1.12	45	1.91
Total	56	3.08	27	5.04	83	3.53
**Customer Service**						
Negative Interaction	5	0.28	15	2.80	20	0.85
Positive Interaction	52	2.86	7	1.31	59	2.51
Total	57	3.14	22	4.10	79	3.36
**Behavioral Response**						
Altered pet care and/or husbandry	15	0.83	4	0.75	19	0.81
Other	6	0.33	0	0.00	6	0.26
Shared Results with Someone	24	1.32	3	0.56	27	1.15
Total	45	2.48	7	1.31	52	2.21
**Needs Improvement**	22	1.21	9	1.68	31	1.32
**Peace of Mind**	24	1.32	5	0.93	29	1.23
**Tested Company Accuracy**	10	0.55	7	1.31	17	0.72

### Pet genetic company websites

The frequency of each theme for pet genetic company websites are provided in Table 5. The primary themes identified for each group of genetic companies were: Type of Results, Information, and Trust. However, the frequency count patterns suggested that potential industry leaders presented more content about the type of test results (e.g., health) in the genetic tests they market with information (e.g., purchasing) as a close second. This pattern was reversed for the other companies: their content primarily focused on information, with the type of results they offered as second (Table 5).

#### Primary themes for all genetic company webpages

The most common themes overall on genetic company webpages were related to product information (*N* = 68), services (*N* = 61), and purchasing information (*N* = 59). The type of test results each company could provide was the second most common theme. The most frequent test result advertised was health-related (*N* = 81), followed by breed results (*N* = 62). An example from Wisdom Panel’s homepage read:

The most comprehensive dog DNA test available. Screens for 200+ genetic conditions, 350+ breeds, types and varieties, and 35+ traits.

Following the type of services and tests offered were themes related to trust and accreditation. All companies gave general statements about the technology and methods (*N* = 45) behind their pet genetic tests. For example, Embark’s homepage stated, “Embark can sniff out breeds that make up as little as 5% of your dog’s overall DNA breed mix.” This theme was also related to trustworthiness (*N* = 36; e.g., “Results you can trust”, Embark), endorsements (*N* = 30; e.g., “What Pet Owners say About Us”, Orivet Genetic Pet Care), and credentials (*N* = 11; e.g., “Embark’s test was developed by veterinarians in partnership with Cornell University”). Of interest, this analysis found a particular company’s technology and credentials were directly tied to another pet DTC genetic company:

Powered by Wisdom Panel, Optimal Selection Canine provides breeders with the most comprehensive test of its kind. We’ve taken the latest scientific research on dog population genetics and developed a simple and easy at–home swab test that screens for multiple diseases and traits while also evaluating genetic diversity. This kind of testing can provide advantages over traditional techniques such as pedigree analysis and help breeders to develop proactive, sustainable breeding programs.

Other companies promoted their technologies and methods as continuously under refinement as demonstrated in this statement from Basepaws’ test product page:

When you join Basepaws, you are joining a community that is constantly evolving. Our database grows larger on a daily basis, so when we have updates for your cat’s report, you’ll have them too.

Another theme of importance was the use of targeted statements towards pet-owners such as the following quote from Wisdom Panel’s homepage:

Over 2 million pet parents just like you have chosen Wisdom Panel products to help them take care of their dog for years to come.

Collectively, these statements were most often advertising consumers would learn about their pet (*N* = 55) by using their genetic test or the product would promote pet and owner well-being (*N* = 37). Another quote from Wisdom Panel’s homepage read:

How much do you really know about your pup’s breeds and genetic health? Plan better, care smarter, and love longer with the world’s leading dog DNA test.

This example shows both themes of “Learn About Pet” and “Well-Being.” Other examples included simple statements like “Get to know your cat, inside and out (Basepaws)” and “Do you want to discover more about what makes your furry companion the way they are (EasyDNA)”.

### Consumer reviews

**Fig 1:**
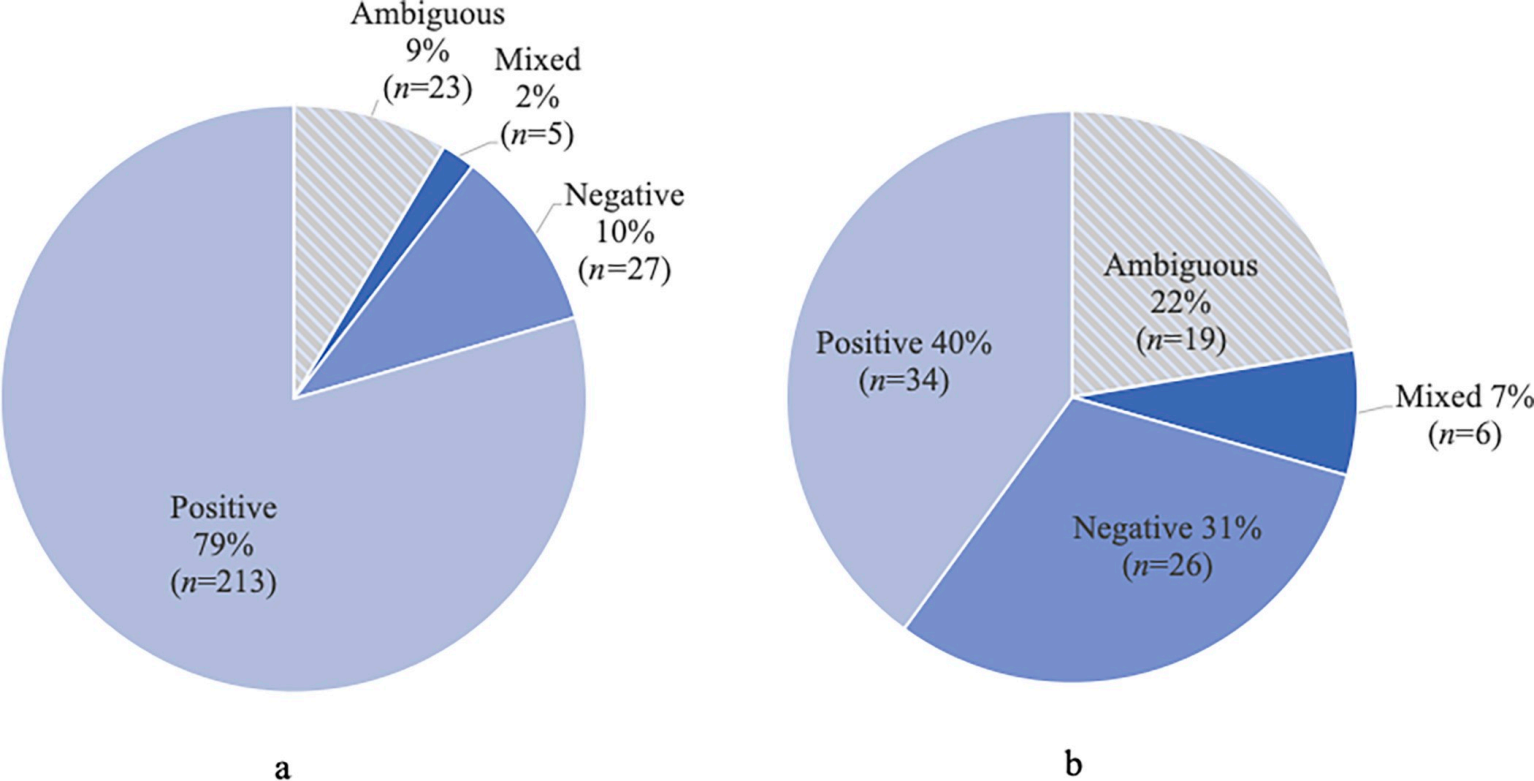
Overall sentiment towards pet DTC-GT available through Amazon. (a) shows the breakdown of consumer sentiments for the companies with more consumer reviews (Basepaws, Embark, Wisdom Panel) and (b) represents consumer sentiments for the remaining companies (Canine HealthCheck, DNA My Dog, Orivet Genetic Pet Care).

As mentioned in the methods, data collection and analysis for consumer reviews were limited by the availability of reviews. No consumer reviews were available from Optimal Selection users as these tests were exclusively sold through their website, though closer review indicated Optimal Selection is associated with Wisdom Panel. Therefore, this section begins with a differentiation between companies with more consumer reviews (Basepaws, Embark, Wisdom Panel) and those with limited amounts (Canine HealthCheck, DNA My Dog, Orivet Genetic Pet Care). As shown in Fig 1, most consumer reviews for both company groups were positive (79% for companies with more reviews and 40% with less reviews), though companies with less reviews had more negative (31%) and ambiguous (22%) sentiments. Two reviews were determined to be irrelevant as the contents of the review were not related to the company or genetic test and, therefore, were excluded from analysis. As it relates to Basepaws, Embark, and Wisdom Panel, when consumers recommended a product in their review, they most often were recommending the company they were reviewing (*n* = 75) rather than another company they were familiar with (*n* = 4). Of note, 5 of the reviewers for the companies with higher review number and 42 of the reviewers for the companies with less consumer reviews were labeled to indicate the person posting the review received a reward for doing so.

Table 6 presents frequencies for each theme identified in consumer reviews and are grouped by the companies and their respective consumer sample size. Although consumer reviews are biased towards Basepaws, Embark, and Wisdom Panel, the primary themes were the same: Perception, Interpretation, and Consumer Experience.

#### Primary themes for all consumer reviews

Of the subthemes related to perception, consumers frequently articulated the test results were informative (*N* = 171) and/or they viewed the test as accurate (*N* = 99). In addition to consumers commenting on their understanding or belief in the test’s accuracy, a small portion of consumers indicated they had also tested the company’s accuracy (*N* = 17). Consumers who indicated they tested the company’s accuracy did so by either not providing all of their pet’s information (e.g., “When I was filling out the information, I purposely left off his breed info [sic] because I didn’t want to give any clues as to what he was.” Embark); tested the same pet more than once using the same company (e.g., “Tested same cat twice, different results.” Basepaws) or used a different company (e.g., a reviewer for Orivet said, “…the breed test was in and it matched Wisdom Panel exactly.”).

The next prevalent theme was associated with how consumers interpreted the test results. Overall, pet owners indicated they used the results to interpret their pet’s breed (*N* = 210) or health (*N* = 131). An example of a consumer using the test to interpret their pet’s breed was:

It delivered the results I expected plus answered questions. She is clearly Great Pyrenees mix but I was never sure of the “mix”. After researching the additional breeds the test returned, I can tell it is extremely accurate. I can now answer when people ask what she’s a mix with. I had previously used another DNA testing brand and it returned pure bred Great Pyrenees which I know was wrong because I know both parents. I was extremely pleased when Wisdom Panel showed the true results. [Wisdom Panel Review]

Although health was the second most common test interpretation, it was often expressed as an added benefit, as compared to the primary mode of interpretation. For example, this quote is from a DNA My Dog review:

Worked great. You should know that if you have a mixed breed like me, your results will come back in percentage ranges, such as 20%-36% Great Pyrenees… .Not only did I receive 5 breeds and the percentage of each my dog might be, the company provided a good bit of info about each breed and their temperaments. Explained a lot lol. And they provided potential health issues my dog might be susceptible to, such as hip dysplaysia [sic], and for each breed. I learned that my "bloodhound mix" was really 2/3 two different breeds of coon hound, and had some St. Bernard in him too. No wonder he’s huge!

Another recurring theme was consumers saying the results gave them peace of mind (*N* = 29). One Basepaws’ consumer said, “It’s also a real blessing know [sic] he has ‘NO’ [sic] inherited Health [sic] issues to be concerned about!’ An Embark review read:

It is wonderful peace of mind knowing my sweet Stella is a healthy well bred [sic] Frenchie and what showed on her test, I can make changes now to avoid. 100% worth 8t [sic]

The third major theme was consumers’ experience with completing a genetic test for their pet. Reviewers commonly stated the test or a process related to the test (e.g., using the website) was easy (N = 165). One consumer commented “Easy to understand (Canine HealthCheck)!” and another said “The test was simple to use and my cat actually let me do it! (Basepaws).” In addition to commenting on the ease of use, other consumers expressed their experience was “fun” (*N* = 77).

## Discussion

Our results showed that companies selling DTC pet genetic tests were primarily advertising product and purchasing information on their webpages. Of the types of products and services offered, genetic companies promoted tests that return health-related results the most, with breed results as their second focus. Themes related to the company’s trustworthiness and credentials appeared to establish the merit of these test results. Building credibility with potential customers is important as experts have expressed concerns about the DTC-GT industry’s lack of transparency and validation in their methods used to derive genetic test results [5, 26].

Another strategy identified in our analysis was genetic companies using statements directed towards pet owners that are suggestive of both pets and “pet parents” benefiting from the test results. These benefits were most associated with the claim that pet owners using a company’s genetic test will know their pet better and pets will, in turn, live healthier and happier lives. This approach can be effective as it relates to how people identify with their pets. By exploring affective human-animal relationships, Charles [27] found that pet owners experience emotional closeness with their pets and understand their relationship in terms of kinship. Previous research has also shown how companion animals can be an extension of their owner’s identity (e.g., [28]). These connections between pet owners and their pets has led to marketing industries targeting human-animal relationships by highlighting the “value” pets bring to consumers [29].

By evaluating the pet owner’s viewpoint of using DTC-GT for their pet(s), we found that Amazon reviews were generally positive. Consumers perceived the test results as accurate and informative. Review comments most often indicated they were being used to determine, or confirm, a pet’s breed identity. Historically, breed classification was utilized by hobbyists and kennel clubs [18]. The question then becomes why are pet owners, who are not “dog enthusiasts” or kennel club members, attentive to their pet’s breed status?

As previously discussed, domestic dog breeds have been created through intense artificial selection, creating an array of phenotypes in dogs. These phenotypes include both physical features (e.g., long vs. short fur, small vs. large body size) and behavioral characteristics. In conjunction with determining their pet’s breed, pet owners also discussed using test results to infer other types of results, which were most related to physical trait status (e.g., carrier for a particular coat color) or behavior (see Table 6). The use of DTC-GT to make behavior or personality inferences, as marketed on company webpages and identified in consumer reviews, merits special consideration.

Extensive research has explored the heritability patterns of canine behavior, with MacLean and colleagues [30] finding a genetic component related to gene-expression of neurological processes (e.g., neurogenesis). Zapata et al.’s [31] research also identified genetic markers in domestic dogs associated with specific behavioral patterns. Prior to these studies, van Rooy et al. [32] cautioned against using genetic panels as a behavior diagnostic as they do not consider pleiotropic genes and environmental influences on animal behavior. For example, Zapata et al. [33] reported gene-environment relationships between behavioral phenotypes and dogs cohabitating with children. Puurunen and colleagues [34] also found environmental factors (e.g., urban vs. rural), owner demographics (e.g., age), dog demographics (e.g., spay or neuter status), and the history of the pet dog’s socialization influenced the expression of fear-related dog behavior.

Although the use of DTC-GT to determine breed heritage was advertised on company websites, it was not the predominant test result promoted. DTC-GT companies most frequently promoted the advantages of using their tests to derive important health-related results. This pattern of companies promoting health benefits first and breed identification second was reversed in consumer reviews, with consumers primarily using the tests for breed identification purposes and the health information secondarily. This finding aligns with Ghirlanda and colleagues’ [35] research into the relationship between breed popularity and breed characteristics. Their study found that social influence impacted breed popularity more than health considerations, with inherited health conditions being secondary in a pet owner’s decision to acquire a particular breed of dog. The notion of breed identity may also parallel human use of DTC-GT results to establish heritage (e.g., [36]).

How pet owners interpret their pet’s genetic test results may influence how they respond to those results. Consumer reviews revealed some pet owners had behavioral responses to test results (see Table 6). The primary behavior response was pet owners sharing their pet’s results with someone, most often a veterinarian. Other reviewers indicated they used the results to alter their pet’s care and husbandry. At the time of this review, only two companies offered genetic counselling as a service with the purchase of a pet DTC-GT. Genetic education counsellors provide support and educational resources to consumers following genetic testing [5]. Kaufman et al. [13] described the role of genetic counsellors as to assist people in interpreting results to prevent unnecessary health-related behavioral responses (e.g., spending money on needless medications). While smaller percentages of the reviews were negative, most often these negative reviews were indicative of the consumer’s poor understanding of the science. For example, pet owners were upset at “unexpected results” in which they were under the impression their pet was a certain breed. Some consumers were also upset with discrepancies in their dog’s actual age and the “genetic age.” Considering this occurrence coupled with pet owners actively responding to results by changing their pet’s care, genetic counselling may become an increasingly important service to help pet owners interpret DNA results as well as prevent negative outcomes associated with those results.

In addition to providing insight into how consumers interpret and respond to their pet’s genetic test results, we found evidence regarding pet owners’ motivations to pursue DTC-GT. When reviewers shared their reasons for purchasing a DTC-GT for their pet, most indicated it was for educational purposes (see Table 6). Educational motives reflected how consumers interpreted the tests: they did so to learn their pet’s breed or to learn more about a pet for which they had little background (e.g., adopted from a shelter). Early research exploring motivations for people getting their own DNA tested also found non-health related reasons (e.g., [15]), such as consumers seeking tests for education, entertainment, and curiosity purposes [26]. Making the parallel between consumers using DTC-GT with little background information, Lee et al. [36] found the majority of adult adoptees used tests to search for biological family, verify “race,” and learn about their ancestry.

### Limitations and future directions

As highlighted in the methods, our sampling techniques were limited by webpage algorithms. Search engines (e.g., Google) and e-commerce platforms (e.g., Amazon) use algorithms that selectively provide materials to users [24]. Considering the authors are based in the US, other web users in different countries may encounter different DTC-GT companies and user reviews on e-commerce platforms. This is also related to the number of DTC-GT pet genetic companies used in this analysis as at the time of this analysis, only eight companies existed in the US. However, our analysis did reveal that Optimal Selection was associated with Wisdom Panel, which further restricted our sample size. While this is representative of the US market, the generalizability of our results to pet genetic companies outside of the US is limited. The home and test product pages were the only content used for genetic company analysis. Therefore, future research may consider a more in-depth content analysis that incorporates non-US based pet genomic companies and more website data. Additional methods could also directly poll DTC-GT providers and distributers.

Another limitation we must acknowledge is our consumer review sample is biased towards companies that are more established and have a larger consumer network. To best account for this, we only used Amazon consumer reviews as compared to other e-commerce platforms (e.g., Chewy.com) as it was the only platform with purchase options for all tests that were not exclusively sold by the company. In addition to standardizing the e-commerce platform for our sample selection, we attempted to standardize the time frames of the consumer reviews by using the 100 most recent reviews for each company. However, these standardizations did not maintain timeline consistency for companies with less than 100 consumer reviews. To acknowledge the sampling biasness, we presented our results as: companies with more consumer reviews, companies with less consumer reviews, and collective frequencies for all companies. The generalizability of our findings to all consumer experiences using pet DTC-GT are limited as we were not able to represent consumer experiences for all of the companies in this analysis and only represent a limited timeframe of consumers using the purchased test.

Another consideration for future research is how e-commerce reviews are limited as consumers may be selective in the information they share and information in reviews are only a “piece of the story.” As it relates to passive data collection and using publicly available information, we were unable to confirm the validity of consumer comments. In-depth interviews would account more for consumer experiences, perceptions, and interpretation of results. Therefore, future research may survey or interview pet owners who use (or do not use) genetic testing for their pets. These methods could also account for consumer experiences for pet genetic companies that are not as established (e.g., EasyDNA) as others (e.g., Embark).

Future research should also examine the DTC-GT market’s relevancy to biotechnology advancements. Genomic editing is predicted to impact veterinary medicine and pet ownership [37]. In discussing the applications of gene editing in companion animals, Sohal and colleagues [37] stated,

Work has…been started on improving pet-owner relationships… .Gene editing has the ability to improve the emotional relationship between pet and owner.

Examples included gene editing cats to diminish hunting behaviors and to create “designer companion animals” [37]. Therefore, one must acknowledge pet DTC-GT as being transformative to these biotechnology aspects. Subsequent research should consider collaborative efforts with pet genetic companies to evaluate the future prospects of DTC-GT. This also highlights the value of veterinarian insights into this industry, to include their knowledge base about the technology and role in genetic counseling.

## Concluding remarks

To our knowledge, this analysis is the first to evaluate the pet animal DTC-GT industry by using webpage and consumer review data. Our objectives were to obtain a more holistic understanding of what pet genetic companies are marketing and what experiences consumers are sharing. In doing so and by incorporating a grounded theory approach, we have set the stage to move past theoretical considerations DTC-GT may have on the human-animal bond and begin empirical investigations. As demonstrated in the success of the human DTC-GT market, this industry will likely grow in popularity. The use of DTC-GT has social and ethical implications, especially if test results can be used adversely against pets and their owners or used in advancing gene editing technologies. Likewise, it is possible these tests have benefits to the human-animal relationship that should be further explored. Therefore, it is increasingly important to evaluate the role genetic testing may have in society and on human-animal dynamics.

## References

[1] Ugalmugle S, Swain R. Genetic testing market size will exceed $28.5 bn by 2026. 2020 Feb 26 [Cited 2 Dec 2020]. Available from: https://www.gminsights.com/pressrelease/genetic-testing-market.

[2] Animal genetics market revenue to cross USD 6.4 bn by 2027 [Internet]. Global Market Insights, Inc. 2021 Mar 8 [Cited 25 Mar 2021]. Available from: https://www.globenewswire.com/news-release/2021/03/08/2188474/0/en/Animal-Genetics-Market-revenue-to-cross-USD-6-4-Bn-by-2027-Global-Market-Insights-Inc.html.

[3] About us [Internet]. Wisdom Panel. 2020 [Cited 2 Dec 2020]. Available from: https://www.wisdompanel.com/en-us/about-us.

[4] Zhang S. What vets think of ‘23andMe for dogs: more and more companies are selling DNA-test kits for pets. The Atlantic. 2018 Nov 12 [Cited 3 Dec 2020]. Available from: https://www.theatlantic.com/science/archive/2018/11/vets-dog-dna-test/575152/.

[5] Moses L, Niemi S, Karlsson E. Pet genomics medicine runs wild. Nature. 2018;559: 470–472. doi: 10.1038/d41586-018-05771-0 30046086

[6] Pet industry market size, trends & ownership statistics [Internet]. American Pet Product Association. 2021 [Cited 30 Mar 2021]. Available from: https://www.americanpetproducts.org/press_industrytrends.asp.

[7] Mendez T. How to compete with Dr. Google. Veterinary Business Advisors, Inc. 2015 [Cited 1 Apr 2021]. Available from: http://veterinarybusinessadvisors.com/wp-content/uploads/2016/07/How_to_Compete_with_Dr._Google_June_2015.pdf.

[8] Kogan L, Oxley JA, Hellyer P, Schoenfeld R, Rishniw M. UK pet owners’ use of the internet for online pet health information. Vet Rec. 2018;182(21): 601. doi: 10.1136/vr.104716 29549181

[9] Kogan L, Schoenfeld-Tacher R, Simon A, Viera A. The internet and pet health information: perceptions and behaviors of pet owners and veterinarians. Internet J Vet Med. 2009;8(1). Available from: http://ispub.com/IJVM/8/1/12921.

[10] Volk JO, Felsted KE, Thomas JG, Siren CW. Executive summary of the Bayer veterinary care usage study. J Am Vet Med Assoc. 2011;238(10): 1275–1282. doi: 10.2460/javma.238.10.1275 21568772

[11] Announcing: The top 10 pet toxins! [Internet]. American Society for the Prevention of Cruelty to Animals. 2020 Mar 13 [Cited 1 Apr 2020]. Available from: https://www.aspca.org/news/announcing-top-10-pet-toxins.

[12] Most common pet toxins [Internet]. Nationwide PetHealthZone. 2021 [Cited 1 Apr 2021]. Available from: https://www.petinsurance.com/healthzone/pet-health/pet-toxins/most-common-pet-toxins/.

[13] Kaufman DJ, Bollinger JM, Dvoskin RL, Scott JA. Risky business: risk perception and the use of medical services among customers of DTC personal genetic testing. J Genet Couns. 2012;21(3): 413–422. doi: 10.1007/s10897-012-9483-0 22278220

[14] Ostergren JE, Gornick MC, Carere DA, Kalia SS, Uhlmann WR, Ruffin, MT, et al. How well do customers of direct-to-consumer personal genomic testing services comprehend genetic test results? Findings from the impact of personal genomics study. Public Health Genomics. 2015;18(4): 216–224. doi: 10.1159/000431250 26087778 PMC4926310

[15] Caulfield T, McGuire AL. Direct-to-consumer genetic testing: perceptions, problems, and policy responses. Annu Rev Med. 2012;63: 23–33. doi: 10.1146/annurev-med-062110-123753 21888511

[16] Dog breeds [Internet]. American Kennel Club. 2021 [Cited 2 Apr 2021]. Available from: https://www.akc.org/dog-breeds/.

[17] CFA breeds [Internet]. Cat Fanciers’ Association. 2021 [Cited 2 Apr 2021]. Available from: https://cfa.org/breeds/.

[18] Fiala I. Dog breeds: the canine version of a socially constructed race. Humanit Soc Sci Rev. 2013;2(4): 137–144.

[19] Why breed-specific legislation is not the answer [Internet]. Am Vet Med Assoc. 2021 [Cited 2 Apr 2021]. Available from: https://www.avma.org/resources/pet-owners/why-breed-specific-legislation-not-answer.

[20] Gunter LM, Barber RT, Wynne CD. A canine identity crisis: genetic breed heritage testing of shelter dogs. PLoS ONE. 2018;13(8): e0202633. doi: 10.1371/journal.pone.0202633 30138476 PMC6107223

[21] Guenther KM. “Taking the ghetto out of the dog:” reproducing inequality in pit bull rescue. Ethn Racial Stud. 2020;43(10): 1795–1812. doi: 10.1080/01419870.2019.1665695

[22] Guest G, MacQueen KM, Namey EE. Applied thematic analysis. Thousands Oaks, CA: SAGE Publications, Inc; 2012. doi: 10.4135/9781483384436

[23] Wells K. The best dog DNA test [Internet]. N Y Times. 2021 Feb 22 [Cited 2 Apr 2021]. Available from https://www.nytimes.com/wirecutter/reviews/best-dog-dna-test/.

[24] Brake DR. The invisible hand of the unaccountable algorithm: how Google, Facebook and other tech companies are changing journalism. In: Tong J, Lo S-H, editors. Digital technology and journalism. Springer International Publishing; 2017. pp. 25–46.

[25] RStudio Team. Integrated Development for R [Internet]. RStudio, PBC, Boston, MA. 2020. Available from: http://www.rstudio.com/.

[26] Wagner JK, Weiss KM. Attitudes on DNA ancestry tests. Hum Genet. 2012;131(1): 41–56. doi: 10.1007/s00439-011-1034-5 21698460

[27] Charles N. ‘Animals just love you as you are’: experiencing kinship across the species barrier. Sociol. 2014;48(4): 715–730. doi: 10.1177/0038038513515353

[28] Jyrinki H. Pet‐related consumption as a consumer identity constructor. Int J Consum Stud. 2012;36(1): 114–120.

[29] Amyx DA. The effects of values, advertising characteristics, and animal companion preference on consumer attitudes and purchase. In: Stieler M, editor. Creating marketing magic and innovative future marketing trends. Springer, Cham; 2017. pp. 65–82. doi: 10.1007/978-3-319-45596-9_15

[30] MacLean EL, Snyder-Mackler N, VonHoldt BM, Serpell JA. Highly heritable and functionally relevant breed differences in dog behaviour. Proc Roy Soc B. 2019;286(1912). doi: 10.1098/rspb.2019.0716 31575369 PMC6790757

[31] Zapata I, Serpell JA, Alvarex CE. Genetic mapping of canine fear and aggression. BMC Genomics. 2016. doi: 10.1186/s12864-016-2936-3 27503363 PMC4977763

[32] van Rooy D, Arnott ER, Early JB, McGreevy P, Wade CM. Holding back the genes: Limitations of research into canine behavioural genetics. Canine Genet Epidemiol. 2014;1(7). doi: 10.1186/2052-6687-1-7 26401324 PMC4579367

[33] Zapata I, Lilly ML, Herron ME, Alvarez CE. Genetic testing of dogs predicts problem behaviors in clinical and nonclinical samples. bioRxiv. 2020. doi: 10.1101/2020.08.13.249805 PMC8819838 35130840

[34] Puurunen J, Hakanen E, Salonen MK, Mikkola S, Sulkama S, Araujo C, et al. Inadequate socialisation, inactivity, and urban living environment are associated with social fearfulness in pet dogs. Sci Rep. 2020;10(3527). doi: 10.1038/s41598-020-60546-w 32103117 PMC7044223

[35] Ghirlanda S, Acerbi A, Herzog H, Serpell JA. Fashion vs. function in cultural evolution: the case of breed popularity. PLoS ONE 2013;8(9): e74770. doi: 10.1371/journal.pone.0074770 24040341 PMC3770587

[36] Lee H, Vogel RI, LeRoy B, Zierhut HA. Adult adoptees and their use of direct-to-consumer genetic testing: searching for family, searching for health. J Genet Couns. 2019;30(1): 144–157. doi: 10.1002/jgc4.1304 32602181

[37] Sohal JS, Khan A, Vats D, Jain M, Polavarapu R, Aseri GK, et al. Applications of genome editing in pet world. In: Malik YS, Barh D, Azevedo V, Kurana SMP, editors. Genomics and biotechnological advances in veterinary, poultry, and fisheries. London UK: Academic Press; 2020. pp. 151–162. doi: 10.1016/B978-0-12-816352-8.00006–0

